# Bis(2,3-dimeth­oxy-10-oxostrychni­din­ium) phthalate nonahydrate

**DOI:** 10.1107/S160053681301204X

**Published:** 2013-05-11

**Authors:** P. Krishnan, K. Gayathri, N. Sivakumar, B. Gunasekaran, G. Anbalagan

**Affiliations:** a Department of Physics, Presidency College, Chennai 600 005, India; b Department of Physics & Nano Technology, SRM University, SRM Nagar, Kattankulathur, Kancheepuram Dist., Chennai 603 203, Tamil Nadu, India

## Abstract

The asymmetric unit of the title compound 2C_23_H_27_N_2_O_4_^+^·C_8_H_4_O_4_^2−^·9H_2_O, contains a cation, an anionon a twofold axis and four and half mol­ecules of water, one of which is located on the twofold axis. In the cation, both fused pyrrolidine rings exhibit twisted conformations, while the piperidine rings adopt screw–boat and boat conformations. In the crystal, the components are linked by N—H⋯O and O—H⋯O hydrogen bonds. The brucinium cations form typical undulating head-to-tail ribbon structuresalong the *a*-axis direction, which associate with the carb­oxy phthalate and the water mol­ecules.

## Related literature

For general background to brucine derivatives, see: Smith *et al.* (2006 ▶) and for related structures, see: Smith *et al.* (2005 ▶, 2006 ▶). 
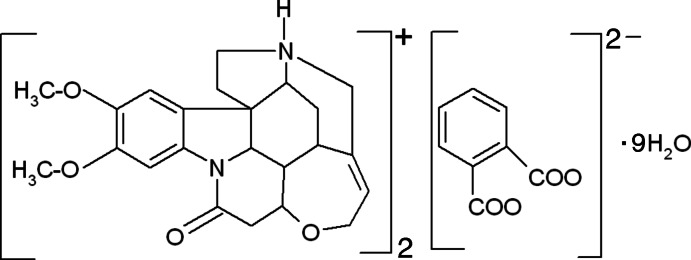


## Experimental

### 

#### Crystal data

2C_23_H_27_N_2_O_4_^+^·C_8_H_4_O_4_^2−^·9H_2_O
*M* *_r_* = 1117.19
Monoclinic, 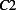

*a* = 13.939 (5) Å
*b* = 12.370 (5) Å
*c* = 15.321 (5) Å
β = 90.646 (5)°
*V* = 2641.6 (17) Å^3^
*Z* = 2
Mo *K*α radiation
μ = 0.11 mm^−1^
*T* = 295 K
0.35 × 0.30 × 0.20 mm

#### Data collection

Bruker APEXII CCD diffractometer
Absorption correction: multi-scan (*SADABS*; Sheldrick, 1996 ▶) *T*_min_ = 0.963, *T*_max_ = 0.979
12452 measured reflections
5094 independent reflections
3854 reflections with *I* > 2σ(*I*)
*R*_int_ = 0.038

#### Refinement

*R*[*F* ^2^ > 2σ(*F* ^2^)] = 0.049
*wR*(*F* ^2^) = 0.125
*S* = 1.05
5094 reflections
385 parameters
16 restraints
H atoms treated by a mixture of independent and constrained refinement
Δρ_max_ = 0.25 e Å^−3^
Δρ_min_ = −0.22 e Å^−3^
Absolute structure: Flack (1983 ▶), 8565 Friedel pairs
Flack parameter: 0 (0)

### 

Data collection: *APEX2* (Bruker, 2004 ▶); cell refinement: *SAINT* (Bruker, 2004 ▶); data reduction: *SAINT*; program(s) used to solve structure: *SHELXS97* (Sheldrick, 2008 ▶); program(s) used to refine structure: *SHELXL97* (Sheldrick, 2008 ▶); molecular graphics: *PLATON* (Spek, 2009 ▶); software used to prepare material for publication: *SHELXL97*.

## Supplementary Material

Crystal structure: contains datablock(s) global, I. DOI: 10.1107/S160053681301204X/rk2399sup1.cif

Structure factors: contains datablock(s) I. DOI: 10.1107/S160053681301204X/rk2399Isup2.hkl

Additional supplementary materials:  crystallographic information; 3D view; checkCIF report

## Figures and Tables

**Table 1:** Hydrogen-bond geometry (Å, °)

*D*—H⋯*A*	*D*—H	H⋯*A*	*D*⋯*A*	*D*—H⋯*A*
N3—H3⋯O5^i^	0.91	1.79	2.665 (4)	159
O7—H7*A*⋯O3	0.82 (1)	1.98 (2)	2.776 (3)	165 (7)
O7—H7*B*⋯O5^ii^	0.82 (1)	2.10 (4)	2.836 (4)	149 (7)
O9—H9*A*⋯O6^iii^	0.82 (1)	1.98 (2)	2.775 (5)	164 (6)
O8—H8*B*⋯O7	0.82 (1)	1.99 (2)	2.794 (5)	165 (6)
O8—H8*A*⋯O9	0.82 (1)	1.97 (3)	2.753 (5)	158 (7)
O9—H9*B*⋯O11^iv^	0.82 (1)	2.01 (1)	2.824 (6)	176 (8)
O10—H10*C*⋯O6^v^	0.82 (1)	2.29 (3)	3.066 (5)	159 (9)
O10—H10*D*⋯O8^vi^	0.82 (1)	1.99 (3)	2.767 (5)	158 (8)
O11—H11*A*⋯O10	0.82 (1)	2.12 (2)	2.808 (4)	142 (3)

Symmetry codes: (i) 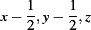; (ii) 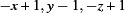; (iii) 
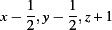
; (iv) 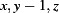; (v) 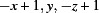; (vi) 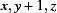.

**Fig. 1.**
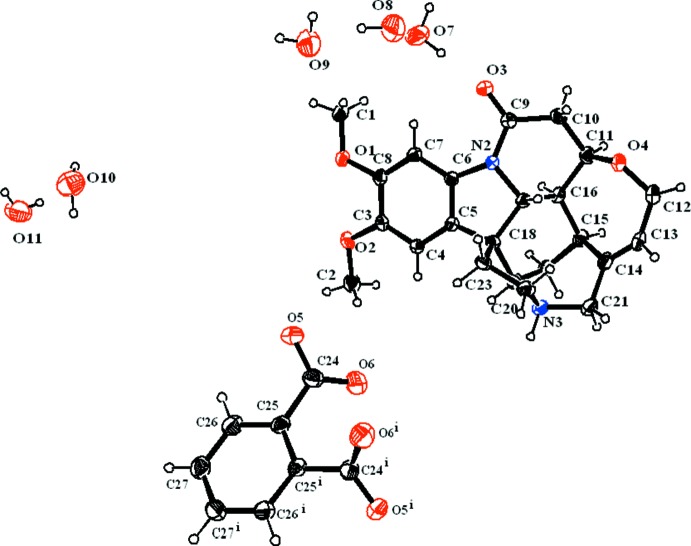
The molecular structure of title compound with the atom labels. Displacement ellipsoids are drawn at 30% probability level. H atoms are presented as a small spheres of arbitrary radius.

## supplementary crystallographic information

### Comment

The strychnos alkaloids, strychnine and brucine have mostly been used to resolve enantiomorphic mixtures of chiral compounds, and the number of crystal structures of both salts and adducts of strychnine (Smith *et al.*, 2006).

The geometric parameters of the title molecule (Fig. 1) agree well with reported similar structure (Smith *et al.*, 2005; 2006). Both fused pyrrolidine rings exhibit twisted conformations, while the piperidine rings adopt screw–boat and boat conformations. In title compound anion placed in special position - twofold axis. One of water molecules placed on twofold axis too. Other four water molecules placed in common positons.

The molecular structure of the compound is stabilized by classical intermolecular N—H···O, O—H···O hydrogen bonds - look Table 1.

### Experimental

The title compound was obtained by dissolving brucine (0.01 mol) in ethanol–water mixture of 50 ml and potassium hydrogen phthalate (0.01 mol) in 10 ml of water. The resulting solution mixed together and stirred well for 1 h. White precipitate has formed and further recrystalized in the mixture of ethanol–water, filtered off, and then allowed to evaporate at room temperature resulting in blocks of crystals within a week.

### Refinement

The C and N bounded H atoms were positioned geometrically and refined using riding model with C—H = 0.93Å and *U*_iso_(H) = 1.2*U*_eq_(C) for aromatic H, C—H = 0.98Å and *U*_iso_(H) = 1.2*U*_eq_(C) for methine H, C—H = 0.97Å and *U*_iso_(H) = 1.2*U*_eq_(C) for methylene H, C—H = 0.96Å and *U*_iso_(H) = 1.5*U*_eq_(C) for methyl H, N—H = 0.91Å and *U*_iso_(H) = 1.5*U*_eq_(N) for amino H. The water H atoms were located from difference Fourier map and refined with using DFIX instruction - O—H = 0.82Å and *U*_iso_(H) = 1.5*U*_eq_(O).

### Crystal data

**Table:**

2C_23_H_27_N_2_O_4_^+^·C_8_H_4_O_4_^2^^−^·9H_2_O	*F*(000) = 1192
*M**_r_* = 1117.19	*D*_x_ = 1.405 Mg m^−^^3^
Monoclinic, *C*2	Mo *K*α radiation, λ = 0.71073 Å
Hall symbol: C 2y	Cell parameters from 5046 reflections
*a* = 13.939 (5) Å	θ = 2.6–26.2°
*b* = 12.370 (5) Å	µ = 0.11 mm^−^^1^
*c* = 15.321 (5) Å	*T* = 295 K
β = 90.646 (5)°	Block, colourless
*V* = 2641.6 (17) Å^3^	0.35 × 0.30 × 0.20 mm
*Z* = 2	

### Data collection

**Table:**

Bruker APEXII CCD diffractometer	5094 independent reflections
Radiation source: fine–focus sealed tube	3854 reflections with *I* > 2σ(*I*)
Graphite monochromator	*R*_int_ = 0.038
Detector resolution: 0 pixels mm^-1^	θ_max_ = 26.2°, θ_min_ = 2.6°
ω– and φ–scans	*h* = −17→16
Absorption correction: multi-scan (*SADABS*; Sheldrick, 1996)	*k* = −14→15
*T*_min_ = 0.963, *T*_max_ = 0.979	*l* = −19→18
12452 measured reflections	

### Refinement

**Table:**

Refinement on *F*^2^	Secondary atom site location: difference Fourier map
Least-squares matrix: full	Hydrogen site location: inferred from neighbouring sites
*R*[*F*^2^ > 2σ(*F*^2^)] = 0.049	H atoms treated by a mixture of independent and constrained refinement
*wR*(*F*^2^) = 0.125	*w* = 1/[σ^2^(*F*_o_^2^) + (0.0613*P*)^2^ + 0.2749*P*] where *P* = (*F*_o_^2^ + 2*F*_c_^2^)/3
*S* = 1.05	(Δ/σ)_max_ < 0.001
5094 reflections	Δρ_max_ = 0.25 e Å^−^^3^
385 parameters	Δρ_min_ = −0.22 e Å^−^^3^
16 restraints	Absolute structure: Flack (1983), 8565 Friedel pairs
Primary atom site location: structure-invariant direct methods	Flack parameter: 0 (0)

### Special details

**Table:**

Geometry. All s.u.'s (except the s.u. in the dihedral angle between two l.s. planes) are estimated using the full covariance matrix. The cell s.u.'s are taken into account individually in the estimation of s.u.'s in distances, angles and torsion angles; correlations between s.u.'s in cell parameters are only used when they are defined by crystal symmetry. An approximate (isotropic) treatment of cell s.u.'s is used for estimating s.u.'s involving l.s. planes.
Refinement. Refinement of *F*^2^ against ALL reflections. The weighted *R*–factor *wR* and goodness of fit *S* are based on *F*^2^, conventional *R*–factors *R* are based on *F*, with *F* set to zero for negative *F*^2^. The threshold expression of *F*^2^ > σ(*F*^2^) is used only for calculating *R*–factors(gt) *etc*. and is not relevant to the choice of reflections for refinement. *R*–factors based on *F*^2^ are statistically about twice as large as those based on *F*, and *R*–factors based on ALL data will be even larger.

### Fractional atomic coordinates and isotropic or equivalent isotropic displacement parameters (Å^2^)

**Table:**

	*x*	*y*	*z*	*U*_iso_*/*U*_eq_	
C1	0.4573 (3)	0.4377 (3)	0.6912 (2)	0.0557 (9)	
H1A	0.4806	0.4845	0.7367	0.084*	
H1B	0.5081	0.3909	0.6727	0.084*	
H1C	0.4052	0.3950	0.7128	0.084*	
C2	0.3724 (3)	0.6817 (3)	0.4106 (2)	0.0599 (10)	
H2A	0.3867	0.7556	0.4250	0.090*	
H2B	0.3083	0.6769	0.3874	0.090*	
H2C	0.4169	0.6564	0.3678	0.090*	
C3	0.36214 (19)	0.5095 (2)	0.47760 (18)	0.0345 (6)	
C4	0.31895 (19)	0.4632 (2)	0.40565 (17)	0.0334 (6)	
H4	0.2979	0.5060	0.3593	0.040*	
C5	0.30711 (18)	0.3514 (2)	0.40293 (16)	0.0308 (6)	
C6	0.33922 (18)	0.2877 (2)	0.47136 (16)	0.0306 (6)	
C7	0.37904 (18)	0.3340 (3)	0.54655 (18)	0.0344 (7)	
H7	0.3984	0.2912	0.5934	0.041*	
C8	0.38888 (19)	0.4445 (3)	0.54950 (17)	0.0354 (7)	
C9	0.37303 (18)	0.0925 (3)	0.48887 (17)	0.0353 (7)	
C10	0.3762 (2)	−0.0096 (3)	0.43528 (19)	0.0389 (7)	
H10A	0.3199	−0.0520	0.4493	0.047*	
H10B	0.4317	−0.0508	0.4546	0.047*	
C11	0.3806 (2)	−0.0001 (3)	0.33534 (19)	0.0410 (7)	
H11	0.4427	−0.0271	0.3154	0.049*	
C12	0.3027 (3)	−0.0866 (3)	0.2121 (2)	0.0615 (10)	
H12A	0.2765	−0.1576	0.1995	0.074*	
H12B	0.3672	−0.0837	0.1890	0.074*	
C13	0.2424 (3)	−0.0029 (3)	0.1685 (2)	0.0512 (9)	
H13	0.1863	−0.0247	0.1403	0.061*	
C14	0.2645 (2)	0.1004 (3)	0.16783 (18)	0.0437 (7)	
C15	0.3573 (2)	0.1421 (3)	0.20946 (18)	0.0413 (7)	
H15	0.4118	0.1105	0.1786	0.050*	
C16	0.36884 (19)	0.1182 (3)	0.30714 (17)	0.0349 (6)	
H16	0.4278	0.1551	0.3259	0.042*	
C17	0.28898 (19)	0.1669 (2)	0.36045 (16)	0.0317 (6)	
H17	0.2336	0.1181	0.3599	0.038*	
C18	0.25598 (19)	0.2826 (2)	0.33607 (16)	0.0311 (6)	
C19	0.2720 (2)	0.3128 (3)	0.24005 (17)	0.0379 (7)	
H19	0.2755	0.3917	0.2353	0.046*	
N3	0.18192 (18)	0.2734 (2)	0.19153 (15)	0.0424 (6)	
H3	0.1573	0.3305	0.1612	0.051*	
C21	0.2013 (2)	0.1850 (3)	0.1277 (2)	0.0497 (8)	
H21A	0.2321	0.2149	0.0767	0.060*	
H21B	0.1411	0.1525	0.1091	0.060*	
C22	0.3610 (2)	0.2643 (3)	0.19939 (18)	0.0432 (7)	
H22A	0.4180	0.2926	0.2281	0.052*	
H22B	0.3635	0.2833	0.1380	0.052*	
C23	0.1456 (2)	0.2932 (3)	0.34231 (19)	0.0375 (7)	
H23A	0.1216	0.2552	0.3929	0.045*	
H23B	0.1264	0.3685	0.3455	0.045*	
C24	0.5857 (2)	0.9656 (3)	0.0600 (2)	0.0449 (8)	
C25	0.5393 (2)	1.0673 (3)	0.02828 (18)	0.0390 (7)	
C26	0.5779 (2)	1.1653 (3)	0.0557 (2)	0.0507 (8)	
H26	0.6305	1.1656	0.0936	0.061*	
C27	0.5394 (3)	1.2624 (3)	0.0275 (2)	0.0566 (9)	
H27	0.5666	1.3274	0.0455	0.068*	
N2	0.32705 (15)	0.1775 (2)	0.45108 (13)	0.0315 (5)	
C20	0.1103 (2)	0.2419 (3)	0.2591 (2)	0.0427 (7)	
H20A	0.1073	0.1640	0.2651	0.051*	
H20B	0.0469	0.2687	0.2435	0.051*	
O1	0.42494 (14)	0.50057 (18)	0.61970 (13)	0.0438 (5)	
O2	0.38030 (15)	0.61733 (18)	0.48644 (14)	0.0448 (5)	
O3	0.40961 (14)	0.09733 (18)	0.56238 (12)	0.0441 (5)	
O4	0.30645 (17)	−0.07057 (18)	0.30469 (13)	0.0496 (6)	
O5	0.5988 (2)	0.9574 (2)	0.14108 (16)	0.0671 (7)	
O6	0.6140 (2)	0.8986 (2)	0.00662 (17)	0.0745 (8)	
O7	0.3608 (3)	0.1234 (3)	0.73624 (18)	0.0916 (10)	
O8	0.1780 (3)	0.0956 (3)	0.8059 (2)	0.1011 (11)	
O9	0.0584 (3)	0.2296 (3)	0.8982 (2)	0.0998 (11)	
O10	0.1784 (2)	0.9654 (4)	0.9528 (2)	0.1013 (11)	
O11	0.0000	1.0515 (4)	1.0000	0.138 (2)	
H7A	0.368 (5)	0.125 (6)	0.6833 (7)	0.208*	
H7B	0.385 (5)	0.068 (4)	0.756 (4)	0.208*	
H9A	0.086 (4)	0.275 (4)	0.928 (4)	0.208*	
H8B	0.230 (2)	0.115 (5)	0.788 (4)	0.208*	
H8A	0.156 (5)	0.143 (3)	0.838 (3)	0.208*	
H9B	0.044 (6)	0.178 (4)	0.929 (4)	0.208*	
H10C	0.2360 (12)	0.966 (8)	0.965 (5)	0.208*	
H10D	0.170 (5)	0.990 (6)	0.904 (2)	0.208*	
H11A	0.0337 (18)	1.0130 (4)	0.9694 (17)	0.208*	

### Atomic displacement parameters (Å^2^)

**Table:**

	*U*^11^	*U*^22^	*U*^33^	*U*^12^	*U*^13^	*U*^23^
C1	0.065 (2)	0.060 (2)	0.0419 (18)	0.0067 (19)	−0.0164 (15)	−0.0125 (16)
C2	0.089 (3)	0.0374 (19)	0.053 (2)	−0.006 (2)	0.0058 (19)	−0.0007 (16)
C3	0.0358 (14)	0.0308 (16)	0.0371 (15)	0.0028 (13)	0.0030 (12)	−0.0071 (12)
C4	0.0394 (15)	0.0308 (17)	0.0299 (14)	0.0006 (13)	0.0000 (11)	0.0018 (11)
C5	0.0330 (13)	0.0306 (15)	0.0287 (14)	0.0016 (13)	0.0004 (11)	−0.0013 (11)
C6	0.0313 (13)	0.0328 (17)	0.0277 (13)	0.0010 (13)	0.0040 (11)	0.0015 (12)
C7	0.0354 (14)	0.0371 (18)	0.0307 (15)	0.0050 (13)	−0.0027 (11)	−0.0011 (11)
C8	0.0314 (14)	0.0403 (18)	0.0343 (15)	0.0024 (13)	−0.0007 (11)	−0.0090 (13)
C9	0.0310 (14)	0.0389 (18)	0.0361 (15)	−0.0002 (14)	0.0016 (11)	0.0023 (13)
C10	0.0379 (15)	0.0357 (18)	0.0431 (16)	0.0052 (14)	−0.0037 (13)	0.0009 (13)
C11	0.0400 (15)	0.0428 (18)	0.0402 (16)	0.0064 (15)	0.0046 (13)	−0.0064 (14)
C12	0.093 (3)	0.041 (2)	0.050 (2)	0.003 (2)	−0.0130 (18)	−0.0142 (16)
C13	0.066 (2)	0.049 (2)	0.0383 (17)	0.0003 (18)	−0.0092 (15)	−0.0167 (15)
C14	0.0534 (18)	0.048 (2)	0.0301 (15)	−0.0033 (16)	0.0000 (12)	−0.0068 (13)
C15	0.0461 (16)	0.049 (2)	0.0291 (15)	−0.0006 (15)	0.0088 (12)	−0.0031 (13)
C16	0.0349 (14)	0.0387 (17)	0.0310 (14)	−0.0025 (14)	0.0003 (11)	−0.0061 (12)
C17	0.0343 (13)	0.0313 (16)	0.0296 (14)	−0.0032 (13)	−0.0020 (11)	−0.0010 (11)
C18	0.0371 (14)	0.0296 (16)	0.0266 (13)	0.0032 (13)	0.0018 (10)	0.0024 (11)
C19	0.0493 (16)	0.0354 (16)	0.0291 (14)	−0.0076 (14)	−0.0004 (12)	0.0024 (12)
N3	0.0557 (15)	0.0387 (15)	0.0325 (12)	−0.0011 (13)	−0.0103 (11)	0.0007 (11)
C21	0.063 (2)	0.050 (2)	0.0353 (16)	−0.0042 (17)	−0.0089 (14)	−0.0039 (14)
C22	0.0520 (17)	0.050 (2)	0.0275 (14)	−0.0095 (16)	0.0071 (13)	0.0009 (13)
C23	0.0403 (15)	0.0302 (16)	0.0419 (16)	0.0044 (14)	−0.0023 (12)	0.0040 (13)
C24	0.0468 (18)	0.043 (2)	0.0445 (18)	−0.0048 (16)	−0.0063 (14)	0.0070 (15)
C25	0.0453 (15)	0.0420 (18)	0.0296 (14)	−0.0038 (15)	0.0002 (11)	0.0031 (13)
C26	0.0595 (19)	0.053 (2)	0.0395 (17)	−0.0143 (18)	−0.0048 (14)	0.0007 (15)
C27	0.079 (2)	0.043 (2)	0.0478 (19)	−0.013 (2)	0.0030 (16)	−0.0017 (15)
N2	0.0364 (12)	0.0309 (13)	0.0271 (11)	0.0016 (11)	−0.0013 (9)	−0.0001 (9)
C20	0.0411 (16)	0.0421 (19)	0.0449 (17)	−0.0026 (15)	−0.0062 (13)	0.0058 (13)
O1	0.0488 (11)	0.0422 (12)	0.0403 (11)	0.0083 (11)	−0.0124 (9)	−0.0118 (9)
O2	0.0557 (13)	0.0271 (12)	0.0516 (13)	−0.0005 (10)	−0.0033 (10)	−0.0082 (9)
O3	0.0534 (12)	0.0436 (13)	0.0350 (11)	0.0076 (11)	−0.0064 (9)	0.0019 (9)
O4	0.0709 (15)	0.0335 (12)	0.0441 (12)	−0.0035 (11)	−0.0092 (10)	−0.0074 (9)
O5	0.0979 (19)	0.0529 (15)	0.0500 (14)	0.0047 (15)	−0.0229 (13)	0.0063 (12)
O6	0.100 (2)	0.0614 (17)	0.0619 (16)	0.0274 (16)	0.0006 (14)	−0.0066 (14)
O7	0.123 (2)	0.107 (3)	0.0452 (15)	0.033 (2)	0.0112 (16)	0.0090 (15)
O8	0.130 (3)	0.090 (2)	0.084 (2)	0.011 (2)	0.0156 (19)	−0.005 (2)
O9	0.111 (3)	0.095 (3)	0.094 (2)	0.023 (2)	−0.007 (2)	−0.0158 (19)
O10	0.096 (2)	0.118 (3)	0.090 (2)	−0.008 (2)	0.0084 (18)	0.009 (2)
O11	0.139 (5)	0.079 (4)	0.199 (7)	0.000	0.076 (4)	0.000

### Geometric parameters (Å, º)

**Table:**

C1—O1	1.413 (4)	C16—C17	1.513 (4)
C1—H1A	0.9600	C16—H16	0.9800
C1—H1B	0.9600	C17—N2	1.487 (3)
C1—H1C	0.9600	C17—C18	1.548 (4)
C2—O2	1.412 (4)	C17—H17	0.9800
C2—H2A	0.9600	C18—C19	1.537 (4)
C2—H2B	0.9600	C18—C23	1.548 (4)
C2—H2C	0.9600	C19—C22	1.517 (4)
C3—O2	1.365 (4)	C19—N3	1.532 (4)
C3—C4	1.375 (4)	C19—H19	0.9800
C3—C8	1.410 (4)	N3—C21	1.494 (4)
C4—C5	1.393 (4)	N3—C20	1.498 (4)
C4—H4	0.9300	N3—H3	0.9100
C5—C6	1.382 (4)	C21—H21A	0.9700
C5—C18	1.505 (4)	C21—H21B	0.9700
C6—C7	1.396 (4)	C22—H22A	0.9700
C6—N2	1.408 (4)	C22—H22B	0.9700
C7—C8	1.374 (4)	C23—C20	1.502 (4)
C7—H7	0.9300	C23—H23A	0.9700
C8—O1	1.371 (3)	C23—H23B	0.9700
C9—O3	1.233 (3)	C24—O6	1.233 (4)
C9—N2	1.358 (4)	C24—O5	1.258 (4)
C9—C10	1.507 (4)	C24—C25	1.493 (4)
C10—C11	1.538 (4)	C25—C25^i^	1.388 (6)
C10—H10A	0.9700	C25—C26	1.390 (4)
C10—H10B	0.9700	C26—C27	1.383 (5)
C11—O4	1.427 (4)	C26—H26	0.9300
C11—C16	1.535 (4)	C27—C27^i^	1.376 (7)
C11—H11	0.9800	C27—H27	0.9300
C12—O4	1.433 (4)	C20—H20A	0.9700
C12—C13	1.487 (5)	C20—H20B	0.9700
C12—H12A	0.9700	O7—H7A	0.8201 (11)
C12—H12B	0.9700	O7—H7B	0.8201 (11)
C13—C14	1.313 (5)	O8—H8B	0.8201 (11)
C13—H13	0.9300	O8—H8A	0.8202 (11)
C14—C21	1.496 (5)	O9—H9A	0.8201 (11)
C14—C15	1.526 (4)	O9—H9B	0.8201 (11)
C15—C22	1.520 (4)	O10—H10C	0.8201 (15)
C15—C16	1.532 (4)	O10—H10D	0.8201 (11)
C15—H15	0.9800	O11—H11A	0.8200 (11)
			
O1—C1—H1A	109.5	N2—C17—C18	104.2 (2)
O1—C1—H1B	109.5	C16—C17—C18	117.1 (2)
H1A—C1—H1B	109.5	N2—C17—H17	109.6
O1—C1—H1C	109.5	C16—C17—H17	109.6
H1A—C1—H1C	109.5	C18—C17—H17	109.6
H1B—C1—H1C	109.5	C5—C18—C19	116.2 (2)
O2—C2—H2A	109.5	C5—C18—C17	102.8 (2)
O2—C2—H2B	109.5	C19—C18—C17	114.2 (2)
H2A—C2—H2B	109.5	C5—C18—C23	111.9 (2)
O2—C2—H2C	109.5	C19—C18—C23	101.2 (2)
H2A—C2—H2C	109.5	C17—C18—C23	110.9 (2)
H2B—C2—H2C	109.5	C22—C19—N3	110.2 (2)
O2—C3—C4	124.5 (3)	C22—C19—C18	115.2 (2)
O2—C3—C8	115.6 (2)	N3—C19—C18	105.1 (2)
C4—C3—C8	119.9 (3)	C22—C19—H19	108.7
C3—C4—C5	119.2 (3)	N3—C19—H19	108.7
C3—C4—H4	120.4	C18—C19—H19	108.7
C5—C4—H4	120.4	C21—N3—C20	112.9 (2)
C6—C5—C4	120.4 (2)	C21—N3—C19	113.4 (2)
C6—C5—C18	109.9 (2)	C20—N3—C19	107.3 (2)
C4—C5—C18	129.6 (2)	C21—N3—H3	107.7
C5—C6—C7	120.9 (3)	C20—N3—H3	107.7
C5—C6—N2	110.4 (2)	C19—N3—H3	107.7
C7—C6—N2	128.7 (2)	N3—C21—C14	110.7 (2)
C8—C7—C6	118.3 (3)	N3—C21—H21A	109.5
C8—C7—H7	120.9	C14—C21—H21A	109.5
C6—C7—H7	120.9	N3—C21—H21B	109.5
O1—C8—C7	124.4 (3)	C14—C21—H21B	109.5
O1—C8—C3	114.6 (3)	H21A—C21—H21B	108.1
C7—C8—C3	121.0 (2)	C19—C22—C15	108.8 (2)
O3—C9—N2	122.7 (3)	C19—C22—H22A	109.9
O3—C9—C10	121.6 (3)	C15—C22—H22A	109.9
N2—C9—C10	115.7 (2)	C19—C22—H22B	109.9
C9—C10—C11	118.7 (3)	C15—C22—H22B	109.9
C9—C10—H10A	107.6	H22A—C22—H22B	108.3
C11—C10—H10A	107.6	C20—C23—C18	103.2 (2)
C9—C10—H10B	107.6	C20—C23—H23A	111.1
C11—C10—H10B	107.6	C18—C23—H23A	111.1
H10A—C10—H10B	107.1	C20—C23—H23B	111.1
O4—C11—C16	114.5 (2)	C18—C23—H23B	111.1
O4—C11—C10	104.2 (2)	H23A—C23—H23B	109.1
C16—C11—C10	110.3 (2)	O6—C24—O5	123.8 (3)
O4—C11—H11	109.2	O6—C24—C25	119.5 (3)
C16—C11—H11	109.2	O5—C24—C25	116.6 (3)
C10—C11—H11	109.2	C25^i^—C25—C26	119.28 (18)
O4—C12—C13	111.2 (3)	C25^i^—C25—C24	122.60 (16)
O4—C12—H12A	109.4	C26—C25—C24	118.1 (3)
C13—C12—H12A	109.4	C27—C26—C25	121.0 (3)
O4—C12—H12B	109.4	C27—C26—H26	119.5
C13—C12—H12B	109.4	C25—C26—H26	119.5
H12A—C12—H12B	108.0	C27^i^—C27—C26	119.7 (2)
C14—C13—C12	123.3 (3)	C27^i^—C27—H27	120.1
C14—C13—H13	118.3	C26—C27—H27	120.1
C12—C13—H13	118.3	C9—N2—C6	126.9 (2)
C13—C14—C21	123.1 (3)	C9—N2—C17	119.5 (2)
C13—C14—C15	121.6 (3)	C6—N2—C17	109.4 (2)
C21—C14—C15	115.3 (3)	N3—C20—C23	105.2 (2)
C22—C15—C14	108.9 (3)	N3—C20—H20A	110.7
C22—C15—C16	106.7 (2)	C23—C20—H20A	110.7
C14—C15—C16	114.9 (2)	N3—C20—H20B	110.7
C22—C15—H15	108.7	C23—C20—H20B	110.7
C14—C15—H15	108.7	H20A—C20—H20B	108.8
C16—C15—H15	108.7	C8—O1—C1	116.2 (3)
C17—C16—C15	112.4 (2)	C3—O2—C2	117.2 (2)
C17—C16—C11	107.7 (2)	C11—O4—C12	115.4 (2)
C15—C16—C11	118.0 (2)	H7A—O7—H7B	108.96 (19)
C17—C16—H16	106.0	H8B—O8—H8A	109.0 (2)
C15—C16—H16	106.0	H9A—O9—H9B	108.96 (19)
C11—C16—H16	106.0	H10C—O10—H10D	109.0 (2)
N2—C17—C16	106.4 (2)		
			
O2—C3—C4—C5	177.8 (3)	C5—C18—C19—C22	85.2 (3)
C8—C3—C4—C5	−3.6 (4)	C17—C18—C19—C22	−34.2 (3)
C3—C4—C5—C6	−0.6 (4)	C23—C18—C19—C22	−153.4 (2)
C3—C4—C5—C18	174.7 (2)	C5—C18—C19—N3	−153.4 (2)
C4—C5—C6—C7	3.7 (4)	C17—C18—C19—N3	87.2 (3)
C18—C5—C6—C7	−172.4 (2)	C23—C18—C19—N3	−32.0 (3)
C4—C5—C6—N2	−175.0 (2)	C22—C19—N3—C21	9.6 (3)
C18—C5—C6—N2	8.9 (3)	C18—C19—N3—C21	−115.0 (3)
C5—C6—C7—C8	−2.4 (4)	C22—C19—N3—C20	134.9 (3)
N2—C6—C7—C8	176.0 (2)	C18—C19—N3—C20	10.3 (3)
C6—C7—C8—O1	178.6 (2)	C20—N3—C21—C14	−76.0 (3)
C6—C7—C8—C3	−1.8 (4)	C19—N3—C21—C14	46.3 (3)
O2—C3—C8—O1	3.1 (3)	C13—C14—C21—N3	126.8 (3)
C4—C3—C8—O1	−175.6 (2)	C15—C14—C21—N3	−52.6 (3)
O2—C3—C8—C7	−176.4 (2)	N3—C19—C22—C15	−62.8 (3)
C4—C3—C8—C7	4.9 (4)	C18—C19—C22—C15	55.8 (3)
O3—C9—C10—C11	−148.1 (3)	C14—C15—C22—C19	56.7 (3)
N2—C9—C10—C11	32.3 (4)	C16—C15—C22—C19	−67.9 (3)
C9—C10—C11—O4	−130.1 (3)	C5—C18—C23—C20	166.8 (2)
C9—C10—C11—C16	−6.7 (4)	C19—C18—C23—C20	42.5 (3)
O4—C12—C13—C14	64.8 (5)	C17—C18—C23—C20	−79.0 (3)
C12—C13—C14—C21	−176.9 (3)	O6—C24—C25—C25^i^	54.8 (5)
C12—C13—C14—C15	2.4 (5)	O5—C24—C25—C25^i^	−129.6 (4)
C13—C14—C15—C22	−178.9 (3)	O6—C24—C25—C26	−124.0 (4)
C21—C14—C15—C22	0.4 (3)	O5—C24—C25—C26	51.5 (4)
C13—C14—C15—C16	−59.3 (4)	C25^i^—C25—C26—C27	0.0 (5)
C21—C14—C15—C16	120.1 (3)	C24—C25—C26—C27	178.8 (3)
C22—C15—C16—C17	61.3 (3)	C25—C26—C27—C27^i^	1.1 (6)
C14—C15—C16—C17	−59.5 (3)	O3—C9—N2—C6	22.6 (4)
C22—C15—C16—C11	−172.4 (3)	C10—C9—N2—C6	−157.8 (2)
C14—C15—C16—C11	66.8 (3)	O3—C9—N2—C17	177.0 (2)
O4—C11—C16—C17	73.9 (3)	C10—C9—N2—C17	−3.4 (3)
C10—C11—C16—C17	−43.2 (3)	C5—C6—N2—C9	159.8 (2)
O4—C11—C16—C15	−54.6 (3)	C7—C6—N2—C9	−18.7 (4)
C10—C11—C16—C15	−171.7 (2)	C5—C6—N2—C17	3.3 (3)
C15—C16—C17—N2	−157.6 (2)	C7—C6—N2—C17	−175.2 (2)
C11—C16—C17—N2	70.8 (3)	C16—C17—N2—C9	−47.6 (3)
C15—C16—C17—C18	−41.6 (3)	C18—C17—N2—C9	−172.0 (2)
C11—C16—C17—C18	−173.2 (2)	C16—C17—N2—C6	110.9 (2)
C6—C5—C18—C19	−142.0 (2)	C18—C17—N2—C6	−13.5 (3)
C4—C5—C18—C19	42.3 (4)	C21—N3—C20—C23	142.2 (2)
C6—C5—C18—C17	−16.6 (3)	C19—N3—C20—C23	16.6 (3)
C4—C5—C18—C17	167.7 (3)	C18—C23—C20—N3	−36.9 (3)
C6—C5—C18—C23	102.5 (3)	C7—C8—O1—C1	2.8 (4)
C4—C5—C18—C23	−73.2 (3)	C3—C8—O1—C1	−176.7 (3)
N2—C17—C18—C5	17.5 (2)	C4—C3—O2—C2	−13.5 (4)
C16—C17—C18—C5	−99.7 (2)	C8—C3—O2—C2	167.8 (3)
N2—C17—C18—C19	144.3 (2)	C16—C11—O4—C12	66.5 (3)
C16—C17—C18—C19	27.1 (3)	C10—C11—O4—C12	−172.8 (2)
N2—C17—C18—C23	−102.2 (2)	C13—C12—O4—C11	−88.0 (4)
C16—C17—C18—C23	140.6 (2)		

Symmetry code: (i) −*x*+1, *y*, −*z*.

### Hydrogen-bond geometry (Å, º)

**Table:**

*D*—H···*A*	*D*—H	H···*A*	*D*···*A*	*D*—H···*A*
N3—H3···O5^ii^	0.91	1.79	2.665 (4)	159
O7—H7*A*···O3	0.82 (1)	1.98 (2)	2.776 (3)	165 (7)
O7—H7*B*···O5^iii^	0.82 (1)	2.10 (4)	2.836 (4)	149 (7)
O9—H9*A*···O6^iv^	0.82 (1)	1.98 (2)	2.775 (5)	164 (6)
O8—H8*B*···O7	0.82 (1)	1.99 (2)	2.794 (5)	165 (6)
O8—H8*A*···O9	0.82 (1)	1.97 (3)	2.753 (5)	158 (7)
O9—H9*B*···O11^v^	0.82 (1)	2.01 (1)	2.824 (6)	176 (8)
O10—H10*C*···O6^vi^	0.82 (1)	2.29 (3)	3.066 (5)	159 (9)
O10—H10*D*···O8^vii^	0.82 (1)	1.99 (3)	2.767 (5)	158 (8)
O11—H11*A*···O10	0.82 (1)	2.12 (2)	2.808 (4)	142 (3)

Symmetry codes: (ii) *x*−1/2, *y*−1/2, *z*; (iii) −*x*+1, *y*−1, −*z*+1; (iv) *x*−1/2, *y*−1/2, *z*+1; (v) *x*, *y*−1, *z*; (vi) −*x*+1, *y*, −*z*+1; (vii) *x*, *y*+1, *z*.
